# Osteogenic Differentiation of Human Umbilical Cord Blood Mesenchymal Stem Cells Induced by Liu's Zhenggudan No. 2 Formula

**DOI:** 10.1155/2022/4718438

**Published:** 2022-09-06

**Authors:** Qiaosong Deng, Shengru Wang, Zheyi Dai, Xiaojun Li, Guanshen Li, Ziheng Wang, Jianfeng Chen

**Affiliations:** ^1^Department of Spine, Wuxi Hospital Affiliated to Nanjing University of Chinese Medicine, Wuxi 214000, China; ^2^Department of Orthopaedic, Changzhou Hospital of Traditional Chinese Medicine, Changzhou, China; ^3^Centre for Precision Medicine Research and Training, Faculty of Health Sciences, University of Macau, Macau SAR, China

## Abstract

**Aim:**

This study aimed to investigate the potential of Liu's Zhenggudan No. 2 Formula (LZF2) in inducing osteogenic differentiation of human umbilical cord blood mesenchymal stem cells (hUCB-MSCs) and treating osteoporosis (OP), thereby providing new methods and ideas for the treatment of OP by traditional Chinese medicine.

**Methods:**

Forty sample rats were equally divided into five groups: high-concentration LZF2, low-concentration LZF2, the *Eucommia ulmoides* (EU) group, the classical osteogenesis induction (COI) group, and the blank control group. Eight rats in each group were routinely housed for 7 days. Subsequently, to induce hUCB-MSCs, drug-containing serum was extracted from the abdominal aorta of rats to prepare the osteogenic induction solution. In addition, alkaline phosphatase (ALP) activity and osteocalcin (OCN) content assays, and alizarin red staining were performed on days 3, 6, 9, and 12 after culture.

**Results:**

After induction of hUCB-MSCs, ALP activity and OCN content increased significantly in the high-concentration LZF2 group. Alizarin red staining also depicted numerous orange-red calcified nodules in rats in the high-concentration LZF2 group.

**Conclusion:**

High concentration of LZF2 can facilitate the differentiation of hUCB-MSCs to promote osteogenesis.

## 1. Introduction

Osteoporosis (OP) is a systemic bone metabolic disease characterized by microstructural destruction of bone tissue and low bone mass that can lead to fracture [1]. Epidemiological surveys in China reveal that OP has become a serious health concern for people above 50 years old, predominantly middle-aged and older women. The overall prevalence of OP in the Chinese population aged >40 years is approximately 54.4%, where 3.2% are aged 40–49 years, 19.2% are >50 years, and 32.0% are >65 years [2]. OP increases susceptibility to multiple fractures and greatly impacts patients' survival and quality of life. Therefore, pharmacological therapies are warranted to alleviate the severity or even cure OP.

Common OP treatments used in clinical practice are not only expensive but also have varying adverse effects, making long-term use of these treatments problematic [3]. Currently, bisphosphonates, calcitonin, denumab, and resyilprofen are the drugs for OP treatment. Bisphosphonates, first introduced in the 1990s, are the safest drugs to treat OP. Bisphosphonates bind to hydroxyapatite in bone, particularly at active bone remodeling sites, and reduce the activity of bone-resorbing osteoclasts. Side effects include flu-like symptoms such as fever, bone, joint, and muscle soreness. Rapid intravenous administration of nitrophosphonates can lead to renal dysfunction or even failure. Common side effects of calcitonin include sweating, flushing, nausea, and local inflammation at the injection site. It could also exhibit nasal discomfort, including rhinitis, nasal mucosal irritation, and nasal bleeding. Denumab inhibits osteoclast activation and development and reduces bone resorption, but vitamin D and calcium should be supplemented before use to avoid hypocalcemia. Raloxifene increases the risk of venous thromboembolic disease and other side effects such as hot flashes, night sweats, and leg cramps [4].

In contrast, traditional Chinese medicine (TCM) offers cost-effective solutions with fewer disadvantages [5]. An example of such a treatment is Liu's Zhenggudan Formula (LZF), which includes LZF1 and LZF2. LZF was first developed by Mr. Liu Bingfu, the founder of the Department of Orthopedics and Traumatology at the Wuxi City Hospital of Traditional Chinese Medicine. The main ingredients of LZF2 are *Rehmannia glutinosa*, *Caesalpinia sappan*, *Syzygium aromaticum*, Costustoot, and *Acacia catechu*, and its effect is to heal fractures. LZF2 demonstrates good therapeutic efficacy in clinical practice. According to a report, an imbalance in bone's osteogenic and osteoclastic capacity can induce OP. Therefore, maintaining a balance between the two is crucial for treating OP [6].

Mesenchymal stem cells (MSCs) are found in the stroma of several organs, such as bone marrow, umbilical cord blood, adipose tissue, muscle, and liver [7, 8]. Under specific conditions, MSCs can regenerate and differentiate into various cell lineages, such as adipocytes, osteoblasts, and chondrocytes [9]. Therefore, MSCs could play a crucial role in the regenerative repair of tissues after injury [10]. Human umbilical cord blood-derived MSCs (hUCB-MSCs) are adult stem cells with multi-directional differentiation potential and have a wide range of clinical applications, primarily in tissue repair for various bone tissue and vascular injuries [11]. In this study, drug-containing sera from LZF2-treated rats were collected to culture hUCB-MSCs *in vitro* and induce their differentiation towards osteogenesis. Subsequently, the capacity of LZF2 to induce osteogenic differentiation of hUCB-MSCs and its possible mechanism in OP treatment was examined. Together, our study explores new avenues and ideas for treating OP using TCMs.

## 2. Materials and Methods

### 2.1. Sample Sources

Forty clean, 4-month-old Sprague Dawley rats, weighing 220 g–260 g, were sourced from the Wuxi Institute of Schistosomiasis Prevention and Control. This study was approved by the ethics committee of Wuxi City Hospital of Traditional Chinese Medicine. Experimental animals were treated as per the Guideline for the Care and Use of Laboratory Animals.

### 2.2. Main Apparatus

The apparatus used in this study included a biosafety cabinet (Suzhou Airtech, Suzhou, China), CO_2_ incubator, centrifuge, and vortex mixer (Thermo, China), cell counter (ADAM™), inverted microscope (Motic), 4°C medical refrigerator and −20°C refrigerator (Panasonic Appliances Cold Chain [Dalian], Dalian, China), −80°C ultra-low temperature refrigerator and electric pipette (Thermo, China), pipette gun (Eppendorf, Shanghai, China), and super thermostatic water bath (Shanghai Boxun Medical Equipment Factory, Shanghai, China).

### 2.3. Experimental Reagents

The reagents used in this study included DMEM-F12 basal medium (Gibco), New Zealand fetal bovine serum (FBS; Gibco), 0.25% Trypsin-EDTA (Gibco), MSC osteogenic induction medium (BI), Alizarin red calcium staining kit (BI), phosphate-buffered saline (PBS; HyClone), high-sugar Dulbecco's Modified Eagle's Medium (DMEM; Gibco), and Hank's balanced salt solution (Contex kds545).

### 2.4. Experimental Consumables

The consumables used in this study included 24-well plates (PLATE, 24WL, TCT, PS, W/LID, S, IND, Corning), cell counting plates (NanoEntek), pipette tips (1–1000 *μ*L) blue color (Axygen), and 5 mL pipettes (Corning).

## 3. Experimental Methods

### 3.1. Extraction, Culture, and Identification of hUCB-MSCs

#### 3.1.1. Acquisition of hUCB Specimens

hUCB specimens were obtained from Wuxi Boyalife Stem Cell Co., Ltd. The specimens were sourced from neonatal donations, consented by mothers who gave birth at full term (exclusions: mothers with infectious diseases such as syphilis and AIDS, newborns with neonatal diseases). The specimens were ethically certified, and mothers and their families signed an informed consent form for specimen collection. About 40–60 mL of UCB was collected from healthy, full-term newborns under aseptic conditions in disposable plastic blood collection bags containing 28 mL of blood preservative solution (main component: sodium citrate) and stored at 4°C. Subsequently, the collected specimens were isolated and cultured within 12 h.

#### 3.1.2. Extraction and Culture of hUCB-MSCs

*(1) In Vitro Isolation and Primary Culture of hUCB-MSCs*. The collected hUCB specimens were mixed with Hank's balanced salt solution in a 1 : 1 ratio. Lymphocyte separation solution was then added to a 50 mL centrifuge tube. The diluted cord blood was then carefully added to the centrifuge tube in a 2 : 1 ratio to avoid mixing the two. Afterward, the samples were centrifuged at 2000 r/min at room temperature to form four layers. The milky white lymphocyte solution in the second layer (annular) was carefully pipetted, washed twice with Hank's balanced salt solution, and centrifuged at 1000 r/min for 10 min at room temperature. The supernatant was discarded in this round. A cell suspension was prepared and inoculated into culture flasks at a density of 1 × 10^6^/mL. Samples were incubated in a cell culture incubator at 37°C with 5% CO_2_ (saturated humidity). After 7 days, the unadhered cells were discarded, and the fluid was changed. After that, the fluid was changed every 3 days. A secondary culture was performed when the cell growth reached 80–90% fusion.

*(2) Secondary Culture of hUCB-MSCs*. Cell growth was observed under an inverted microscope, and secondary culture was performed per the following procedures: the culture medium was aspirated, and the cells were rinsed 2-3 times with PBS buffer. The supernatant was discarded, and 1 mL of 0.25% Trypsin-EDTA (trypsin digestion) solution was added for 15–20 seconds to dissociate the cells from the culture flask. The extent of cell dissociation was observed under an inverted microscope. Once the cell protrusions retracted and the cell gaps increased, and cells started to round, the trypsin digestion solution was removed, and an appropriate amount of DMEM-F12 basal medium containing 15% New Zealand FBS was added to the cells. The cell suspension was then transferred to a centrifuge tube after gently tapping the flask bottom and centrifuged at 1000 r/min for 10 min at room temperature. The supernatant was discarded, and the cells were inoculated into culture flasks, labeled as P1, in a 1 : 2 and further incubated. The complete culture medium was changed after 2 or 3 days during the secondary culture. When the cell growth reached 80%–90% confluency, the cells were re-cultured following the above process and labeled P2. The cells were tested and identified after the third cycle of culture generation.

#### 3.1.3. Detection and Identification of hUCB-MSCs

*(1) Morphological Observation of hUCB-MSCs*. Morphological characteristics of primary and secondary hUCB-MSCs and their proliferation status were observed and recorded daily under an inverted microscope.

*(2) Detecting the Proliferation of hUCB-MSCs Using MTT Colorimetric Assay*. After 72 h incubation, the culture solution was discarded, and the samples were rinsed with PBS buffer. Post rinsing, the samples were mixed with 100 *μ*L of PBS buffer and 10 *μ*L of MTT staining solution (5 mg/mL dissolved in PBS buffer to remove bacteria) and incubated at 37°C for 2 h. Later, the crystals are dissolved by adding 100 *μ*L of 10% SDS and mixing using an oscillator. The absorbance is then read at 570 nm on a microplate reader.

### 3.2. Preparation of Osteogenic Induction Solution

#### 3.2.1. Experimental Groups

A total of 40 rats were divided into the following five groups: the high-concentration LZF2 and low-concentration LZF2 groups, the *Eucommia ulmoides* (EU) group, and the classical osteogenesis induction (COI) group, and the blank control group.

#### 3.2.2. Preparation of Drug-Containing Serum

*(1) High- and Low-Concentration LZF2 Groups*. The LZF2 pills (provided by the drug manufacturing room of the Wuxi City Hospital of Traditional Chinese Medicine) were crushed and added to distilled water to make a mixed suspension. Crude drug doses were determined by calculating the body surface area of humans and animals [9]. Drugs were then administered to rats in the high- and low-concentration LZF2 groups by gavage (once a day for 7 consecutive days) in ratios of 1 : 1 (LZF2 to distilled water ratio), respectively.

*(2) EC Group*. EC granules (produced by Jiangsu Jiangyin Tianjiang Pharmaceutical, 1 g/packet) were dissolved in distilled water to make a suspension. Using the same calculation, rats in the EC group were administered the corresponding crude drug dose by gavage (once a day for 7 consecutive days) in a 1 : 1 ratio (LZF2 to distilled water ratio).

*(3) COI Group and Blank Control Group*. Similarly, rats in the COI and blank control groups were administered the same dose of saline as the EC group by gavage (once a day for 7 consecutive days) at the same time every day.

After the last gavage, rats in each group were anesthetized with an intraperitoneal injection of 3% pentobarbital sodium. Once completely anesthetized, using tissue scissors, a T-shaped incision was made in the abdominal cavity of the rats to separate the organs and locate the abdominal aorta. Blood from the abdominal aorta was harvested using a disposable blood collection needle and centrifuged at 1500 r/min for 10 min to segregate the serum. The extracted serum was inactivated in a 56°C water bath, poured through a microporous filter to extract different drug-containing sera, and stored at −20°C for subsequent use.

#### 3.2.3. Preparation of Osteogenic Induction Solution

TCM-based osteogenic induction solution was prepared as follows: 10% FBS + serum from rats containing different drugs (three drug groups) + high-sugar DMEM medium.

Western medicine-based osteogenic induction solution was prepared as follows: MSC osteogenic induction medium (BI) + serum from rats in the Western medicine induction group + high-sugar DMEM medium.

The blank control induction solution was prepared as follows: 10% FBS + serum from rats in the blank control group + high-sugar DMEM medium.

### 3.3. Induction, Detection, and Screening of Osteogenesis

#### 3.3.1. Solution Reagent Preparation

Before starting the experiment, the trypsin and MSCgo™ Osteogenic Differentiation Medium were prewarmed in a 37°C incubator. The complete medium was prepared according to the Standard Operating Procedure 01-LA-011 for Laboratory Solution Preparation and Processing of Divided Reagents and placed in a 37°C incubator to preheat. Additionally, a high-sugar complete medium containing 10% FBS was prepared by adding 56 mL of FBS to 500 mL of high-sugar DMEM.

#### 3.3.2. Osteogenesis Induction Method

Following *in vitro* amplification, P2 hUCB-MSCs were exposed to different inducers for osteogenic induction. After 10–28 days of induction, the cells were stained using an alizarin red agent and imaged using an inverted biological microscope. The specific steps in induction were as follows: (1) In the logarithmic phase, P1 hUCB-MSCs were placed in T75 culture flasks and digested with 3 mL of trypsin for about 1 min. When cells adopted a round shape, the culture flasks were gently tapped to dislodge the cells from the bottom of the flask. An equal volume of complete medium with trypsin was added to the mixture to terminate the digestion. The cell suspension was then collected into a 15 mL centrifuge tube. (2) The supernatant was discarded after centrifugation at 1400 rpm for 5 min. (3) The cells were resuspended in 10 mL of MSC complete medium. Approximately 100 *μ*L of cell suspension was tested twice for cell counting and viability, and the average was taken. (4) Cells were inoculated in 24-well plates at a density of 2 × 10^4^ cells/cm^2^. (5) Cell suspension was inoculated in 24-well plates (2 wells per group, 0.5 mL per well, 37°C, 5% CO_2_) and incubated at saturated humidity. (6) After the cells reach 80% confluency, the culture medium was removed, and 0.5 mL of the corresponding osteogenic induction solution was added to each group. (7) The samples were incubated in a 37°C incubator with 5% CO_2_. (8) The medium was changed every 2-3 days following the procedure below: the culture medium in the wells was aspirated and then discarded, followed by the addition of 0.5 mL of the corresponding osteogenic induction solution for 10–28 days.

#### 3.3.3. Cell Staining

Cell staining was performed according to the product instructions of the Alizarin Red S Staining Kit. The following procedure was followed for sample fixation: (1) The culture medium was carefully removed from the 24-well cell culture plate; (2) 400 *μ*L PBS (HyClone Clean-up Solution) was added to each well to wash the cell surface; (3) PBS was carefully extracted from each well; (4) 300 *μ*L of fixation solution (BI) was added to each well to cover the entire growth surface; (5) samples were left at the room temperature for 30 min; (6) the fixative solution was carefully aspirated; (7) 300 *μ*L of Wash I solution from the kit was added to each well to wash the cell surface 2-3 times. Sample staining was done as follows: (1) Wash I solution was removed, and 300 *μ*L of staining solution (BI) was added to cover the bottom surface evenly; (2) the cells were stained for 30 min at room temperature; (3) the staining solution was carefully aspirated, and 300 *μ*L of Wash II solution was added from the kit to wash the cells 2-3 times; (4) the Wash II solution was removed, 300 *μ*L of inspection solution was added, and the cells were observed under an inverted microscope. Images were taken to record observations.

#### 3.3.4. Alkaline Phosphatase Activity Assay

On days 3, 6, 9, and 12 after induction, 50 *μ*L each of aspirated cell supernatant, double-distilled water, and phenol standard application solution were added to three centrifuge tubes (EP), labeled as the assay tube, blank tube, and standard tube. Subsequently, 0.5 mL of matrix and buffer solution were added to each tube and heated at 37°C in a water bath for 15 min after thorough mixing. A drop of the 1.5 mL color developer was added to the tubes. Each tube's absorbance (OD) was measured by colorimetry on a violet spectrometer at 520 nm and 0.5 cm optical paths.

#### 3.3.5. Osteocalcin Content Measurement

After induction, osteocalcin (OCN) content was measured on days 3, 6, 9, and 12 using the OCN assay kit. OCN antibody reagent was added to the extracted cell supernatant and incubated at 4°C for 20 h. OCN in the samples reacted competitively with an OCN antibody reagent to reach equilibrium. The isolation reagent was added to the sample mixture and incubated at room temperature for 15 min leading to the formation of a precipitate. After centrifugation at 4°C, the unbound OCN was separated, and the supernatant was discarded. The precipitate was then subjected to radio-counting, and the OCN content in the samples was measured based on the standard curve.

### 3.4. Statistical Analysis

One-way analysis of variance (ANOVA) was performed on the data between groups on days 3, 6, 9, and 12 separately using the SPSS 22.0 statistical software. Wilcoxon signed-rank test was used when the ANOVA conditions were not met. Significance was defined as *P* < 0.05, and evaluated data were expressed as mean ± standard deviation.

## 4. Results

### 4.1. Alkaline Phosphatase Activity

After induction, rats in the high-concentration LZF2 group exhibited a significant increase in alkaline phosphatase (ALP) activity compared with those in the blank control group (*P* < 0.05). No statistically significant difference in ALP activity was observed between the low-concentration LZF2 and the EC group versus the blank group. However, the ALP activity of rats in the high-concentration LZF2 group was weaker than that observed in the COI group (Table 1).

### 4.2. OCN Content

Post induction, the OCN content in the COI and high-concentration LZF2 groups showed a significant increase (*P* < 0.05) compared to the blank control group (Table 2).

### 4.3. Alizarin Red Staining

**Figure 1:**
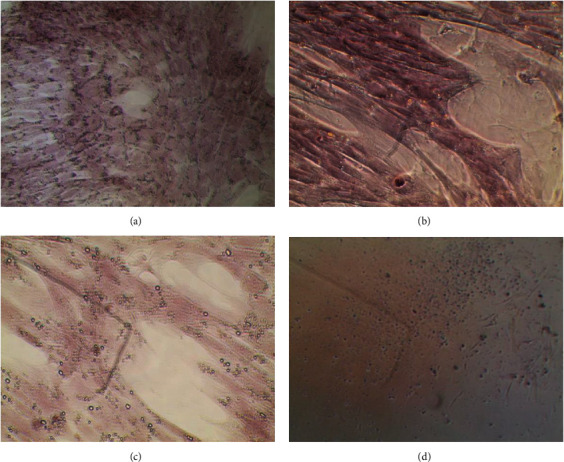
(a) High-concentration LZF2 group D9 (10x); (b) COI group D9 (20x); (c) low-concentration LZF2 group D9 (20x); (d) EC group D9 (20x).

The alizarin red staining in the high- and low-concentration LZF2 groups and the COI group showed the presence of red calcified nodules relative to the EC group. Additionally, it was noted that the high-concentration LZF2 group and the COI group had more red calcified nodules than the low-concentration LZF2 group (Figure 1).

## 5. Summary

Following induction, a significant increase in ALP activity, OCN content, and the number of calcified nodules was observed in the COI and the high-concentration LZF2 groups compared with the blank control group. No statistically significant differences were observed in these assay parameters between the low-concentration LZF2 group and the EC group vs. the blank control group. It is noteworthy that the high-concentration LZF2 group had lower ALP activity and fewer calcified nodules compared with the COI group.

## 6. Discussion

OP, a common geriatric disease, can be categorized as primary and secondary types and is pathologically characterized by thinning bone trabeculae, structural fracture, and reduction in bone mass [12]. Primary OP can occur in men and women of all ages but generally in postmenopausal women. Reduction in postmenopausal ovarian function and declining estrogen levels lead to accelerated bone loss. Osteoporosis and osteopenia in men are considered to be less frequent than in women. In men, OP is more likely to occur later in life, caused by decreased testosterone and estrogen levels. Secondary OP results from drugs (such as glucocorticoids, chemotherapy, etc.) and other diseases (such as hypogonadism, hyperparathyroidism, gastrointestinal diseases, etc.) [13, 14]. Osteoporosis causes more than 9 million fractures annually, about 20 fractures every minute [15]. Studies show that the prevalence of OP in China has increased significantly in the past 12 years, from 14.94% before 2008 to 27.96% between 2012 and 2015. The prevalence of OP was 25.41% in females, and 15.33% in males, and the incidence increased with age. The prevalence of OP in rural areas (20.87%) was higher than that in urban areas (23.92%). Similarly, the prevalence in the southern area (23.17%) was higher than that in the northern area (20.13%). The prevalence of OP in people over 50 is now more than double what it was in 2006 [16]. According to epidemiological statistics, the prevalence of OP in the geriatric population (>60 years) in China is approximately 32%, making OP a major public health concern for the elderly population that endangers their physical health and quality of life [17]. The key to OP treatment is to promote bone formation. hUCB-MSCs have been shown to treat the bone injury by promoting the restoration of vascular blood supply to injured bone tissues. They also facilitate the maintenance of osteogenesis-osteoclasis balance, which is crucial in repairing and reconstructing injured bone tissues and treating orthopedic diseases, including OP. In recent years, traditional Chinese medicine in OP treatment has become a new hotspot [18]. Recent studies have demonstrated that TCM with kidney-tonifying, spleen-tonifying, and stasis-removing effects all have the potential effects of treating OP. The natural products from TCM provide a theoretical basis for clinical application, research, and development of new drugs for osteoporosis [19]. Some examples include Icariin, the main active flavonoid glycoside extracted from the Epimedii Folium; Echinacoside, a phenylethanoid glycoside isolated from Cistanches Herba; Echinoside, isolated from Cistanche Deserticola; and Poncirin, isolated from the fruit of *Poncirus trifoliata*. In the current study, drug-containing sera from LZF2-treated rats induce osteogenic differentiation in hUCB-MSCs which positively affects OP treatment.

TCM theory has classified OP according to its clinical characteristics in the western medical sense as “rheumatism,” “bone fistula,” and “bone impotence,” all of which correspond to the category of impotence. According to the TCM theory, kidney, marrow, and bone are closely related and instrumental in the life cycle of bones: “birth, growth, strength, weakening, and death.” The kidneys store the essence, the essence produces the marrow, the marrow resides in the bones, and the bones grow relying on the bone marrow. When the essence of the kidney is sufficient, the bone marrow has a source of differentiation, resulting in strong and powerful bones. However, the lack of kidney essence leads to a lack of source of bone marrow differentiation and the loss of bone nourishment, resulting in weak, fragile, and fracture-prone bones in the elderly [15]. Therefore, nourishing the liver and tonifying the kidneys are essential for treating OP.

In the current study, sample rats were divided into five groups to investigate the effect of LZF2 in inducing osteogenic differentiation of hUCB-MSCs. ALP is mainly distributed in the cell membrane; it assists transportation of calcium across the membrane and is involved in cell maturation and calcification processes [20]. ALP is not only a marker for early differentiation of osteoblasts but is also necessary for bone tissue mineralization. In this study, elevated ALP activity served as a significant marker of osteogenic differentiation of the hUCB-MSCs. Osteocalcin (OCN) is called bone *γ*-carboxyl glutamate-containing protein. In the process of bone resorption, the carboxyl group on OCN is removed, and decarboxylated OCN is released into the cycle. Therefore, the determination of serum osteocalcin content can reflect the activity of osteoblasts and the degree of bone conversion. Osteoblasts are terminally differentiated cells that secrete osteocalcin. Therefore, osteocalcin is a good marker of bone maturation and late osteogenic differentiation [21].

Alizarin red staining is a critical way to test osteogenic differentiation. During the differentiation of the mesenchymal stem cells to the osteoblasts, calcium salts are deposited on the cell surface, and calcium nodules are formed. Upon staining with alizarin red, calcium ion and alizarin red undergo chelation and produce a dark red color compound. Therefore, osteogenic cells having calcium nodules would stain dark red. The area of staining depicts the extent of the osteogenetic differentiation [22].

MSCs are found in various human tissues and secretions such as adipose tissue, peripheral blood, menstrual blood, endometrium, breast milk, and fetal tissues such as amniotic fluid, membranes, chorionic membrane, placenta, umbilical cord, and umbilical cord blood (UCB) [23]. Under specific conditions, MSCs have the potential to differentiate into multiple cell types such as osteoblasts, chondrocytes, and adipocytes [24, 25]. UCB is abundantly available, is easy and safe to collect, and can be used to isolate and culture MSCs in huge quantities. Moreover, hUCB-MSCs are stable during in vitro culture and are capable of mesodermal cell differentiation. This makes hUCB-MSCs a potentially excellent and reliable source for osteoblasts with the intention of bone tissue repair.

In this study, LZF2 was used to promote the differentiation of hUCB-MSCs to osteoblasts. Results showed a significant increase in ALP activity, OCN content, and the number of mineralized nodules, all of which indicate increased differentiation. However, this study does not clarify how this differentiation of hUCB-MSCs toward osteogenesis is promoted. Therefore, understanding the key signaling pathways and transcription factors involved in this differentiation is essential. TGF-*β*/BMP pathway is the primary signaling cascade of osteogenesis and participates in most osteogenic differentiation processes. Disruption of BMP/TGF-*β*signal transduction leads to multiple diseases. The Wnt pathway affects various cellular activities, such as cell proliferation, growth, differentiation, and cell death. It also plays a vital role in osteoblast differentiation and mineralization. The IGF signaling pathway is also one of the important signaling pathways involved in osteoblast proliferation and differentiation. In addition, transcription factors such as RUNX2, Bmp2, and SOX9 play a vital role in osteoblast differentiation [26]. Our future studies focus on understanding these signaling pathways' involvement in the process of osteogenesis. Overall, this study provides a scientific and objective basis for the clinical application of LZF2 and establishes the significant advantages of TCM in treating orthopedic diseases.

## Figures and Tables

**Table 1:** Alkaline phosphatase activity (OD) at different time points of induction in different groups (mean ± *s*).

Groups	Day 3	Day 6	Day 9	Day 12
Blank control group	0.83 ± 0.11	0.32 ± 0.08	0.49 ± 0.05	0.57 ± 0.23
High-concentration LZF2 group	1.23 ± 0.22^#^	1.24 ± 0.08^#^	0.88 ± 0.09^##^	0.85 ± 0.24^##^
Low-concentration LZF2 group	0.82 ± 0.24	0.56 ± 0.22	0.61 ± 0.07	0.44 ± 0.09
EC group	0.75 ± 0.20	0.28 ± 0.15	0.42 ± 0.08	0.33 ± 0.06
COI group	1.56 ± 0.30^#^	1.49 ± 0.33^#^	1.42 ± 0.05^#^	1.31 ± 0.12^#^

Note: ^#^*P* < 0.05 compared with the blank control group;^##^*P* < 0.05 compared with the COI group.

**Table 2:** Osteocalcin content at different time points after induction in different groups (mean ± *s*).

Groups	Day 3	Day 6	Day 9	Day 12
Blank control group	0.05 ± 0.004	0.08 ± 0.005	0.15 ± 0.012	0.21 ± 0.010
High-concentration LZF2 group	0.11 ± 0.011	0.20 ± 0.050^#^	0.37 ± 0.194^#^	0.42 ± 0.177^#^
Low-concentration LZF2 group	0.06 ± 0.008	0.10 ± 0.006	0.14 ± 0.009	0.23 ± 0.013
EC group	0.08 ± 0.014	0.12 ± 0.007	0.20 ± 0.011	0.25 ± 0.019
COI group	0.17 ± 0.016^#^	0.24 ± 0.017^#^	0.48 ± 0.013^#^	0.52 ± 0.019^#^

Note: ^#^*P* < 0.05 compared with the blank control group.

## Data Availability

The data used to support the findings of this study are included within the article.

## References

[1] Zhao H., Zhao N., Zheng P. (2018). Prevention and treatment of osteoporosis using Chinese medicinal plants: special emphasis on mechanisms of immune modulation. *Journal of Immunology Research*.

[2] Chen S. H., Wang X. L., Zheng L. Z. (2016). Comparative study of two types of herbal capsules with different Epimedium species for the prevention of ovariectomised-induced osteoporosis in rats. *Journal of Orthopaedic Translation*.

[3] Huang J. V., Schooling C. M. (2017). Inflammation and bone mineral density: a Mendelian randomization study. *Scientific Reports*.

[4] Camacho P. M., Petak S. M., Binkley N. (2020). American association of clinical endocrinologists/American college of endocrinology clinical practice guidelines for the diagnosis and treatment of postmenopausal osteoporosis-2020 update. *Endocrine Practice*.

[5] Guo Y. Z., Jiang Y. N., Li Y. F., Kurihara H., Dai Y., He R. R. (2019). Clinical prescription-protein-small molecule-disease strategy (cpsd), A new strategy for Chinese medicine development: a case study in cardiovascular diseases. *Frontiers in Pharmacology*.

[6] He L., Lee J., Jang J. H. (2013). Osteoporosis regulation by salubrinal through eIF2*α* mediated differentiation of osteoclast and osteoblast. *Cellular Signalling*.

[7] Gao Y., Zhao G., Li D., Chen X., Pang J., Ke J. (2014). Isolation and multiple differentiation potential assessment of human gingival mesenchymal stem cells. *International Journal of Molecular Sciences*.

[8] González P. L., Carvajal C., Cuenca J. (2015). Chorion mesenchymal stem cells show superior differentiation, immunosuppressive, and angiogenic potentials in comparison with haploidentical maternal placental cells. *Stem Cells Translational Medicine*.

[9] Zhou H. S., Su X. F., Fu X. L. (2016). Mesenchymal stem cells promote pancreatic adenocarcinoma cells invasion by transforming growth factor-*β*1 induced epithelial-mesenchymal transition. *Oncotarget*.

[10] Liu B., Hu D., Zhou Y. (2020). Exosomes released by human umbilical cord mesenchymal stem cells protect against renal interstitial fibrosis through ROS-mediated P38MAPK/ERK signaling pathway. *American Journal of Tourism Research*.

[11] Kim H. S., Lee J. H., Roh K. H., Jun H. J., Kang K. S., Kim T. Y. (2017). Clinical trial of human umbilical cord blood-derived stem cells for the treatment of moderate-to-severe atopic dermatitis: phase I/IIa studies. *Stem Cells*.

[12] Chin K. Y., Ima-Nirwana S. (2019). The role of tocotrienol in preventing male osteoporosis-A review of current evidence. *International Journal of Molecular Sciences*.

[13] Yong E. L., Logan S. (2021). Menopausal osteoporosis: screening, prevention and treatment. *Singapore Medical Journal*.

[14] NIH Consensus Development Panel on Osteoporosis Prevention D Therapy (2001). Osteoporosis prevention, diagnosis, and therapy. *JAMA*.

[15] Hernlund E., Svedbom A., Ivergard M. (2013). Osteoporosis in the European Union: medical management, epidemiology and economic burden. *Archives of Osteoporosis*.

[16] Chen P., Li Z., Hu Y. (2016). Prevalence of osteoporosis in China: a meta-analysis and systematic review. *BMC Public Health*.

[17] Clynes M. A., Harvey N. C., Curtis E. M., Fuggle N. R., Dennison E. M., Cooper C. (2020). The epidemiology of osteoporosis. *British Medical Bulletin*.

[18] Chen Q., Zeng J., Chen Y. (2019). Efficacy of Xianling Gubao capsule in treating sarco-osteopenia. *Medicine*.

[19] An J., Yang H., Zhang Q. (2016). Natural products for treatment of osteoporosis: the effects and mechanisms on promoting osteoblast-mediated bone formation. *Life Sciences*.

[20] Howlett C. R., Cavé J., Williamson M. (1986). Mineralization in in vitro cultures of rabbit marrow stromal cells. *Clinical Orthopaedics and Related Research*.

[21] Manolagas S. C. (2020). Osteocalcin promotes bone mineralization but is not a hormone. *PLoS Genetics*.

[22] Puchtler H., Meloan S. N., Terry M. S. (1969). On the history and mechanism of alizarin and alizarin red S stains for calcium. *Journal of Histochemistry and Cytochemistry*.

[23] Katz A. J., Tholpady A., Tholpady S. S., Shang H., Ogle R. C. (2005). Cell surface and transcriptional characterization of human adipose‐derived adherent stromal (hADAS) cells. *Stem Cells*.

[24] Zhang Y. L., Liu L., Peymanfar Y., Anderson P., Xian C. J. (2021). Roles of MicroRNAs in osteogenesis or adipogenesis differentiation of bone marrow stromal progenitor cells. *International Journal of Molecular Sciences*.

[25] Hartleif S., Schumm M., Döring M. (2017). Safety and tolerance of donor-derived mesenchymal stem cells in pediatric living-donor liver transplantation: the MYSTEP1 study. *Stem Cells International*.

[26] Hayrapetyan A., Jansen J. A., van den Beucken J. J. (2015). Signaling pathways involved in osteogenesis and their application for bone regenerative medicine. *Tissue Engineering Part B Reviews*.

